# Supplementary figures and images for: Identification of powdery mildew resistance QTL in strawberry (Fragaria × ananassa)
Source: Theor Appl Genet. 2018 Jul 3;131(9):1995–2007. doi: 10.1007/s00122-018-3128-0 (PMC6096635; doi:10.1007/s00122-018-3128-0)

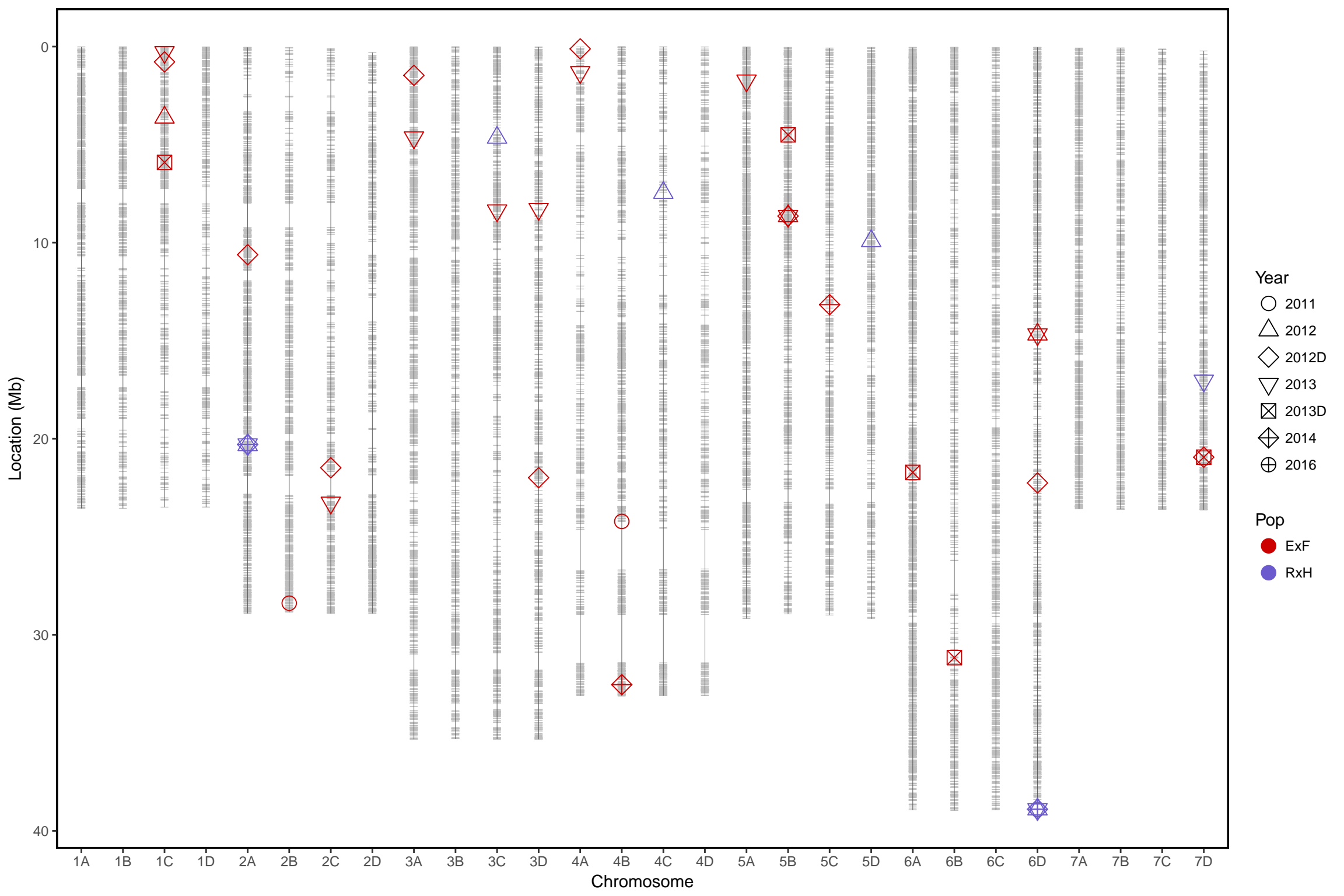

Supplement: Supplementary file 1 — Linkage map displaying 35154 marker positions (grey) in Mb for 28 linkage groups of octoploid strawberry (1A–7D) marker positions scaled to F. vesca genome. QTL locations from each phenotyping event represented (PDF 207 kb) [file 122_2018_3128_MOESM1_ESM.pdf]

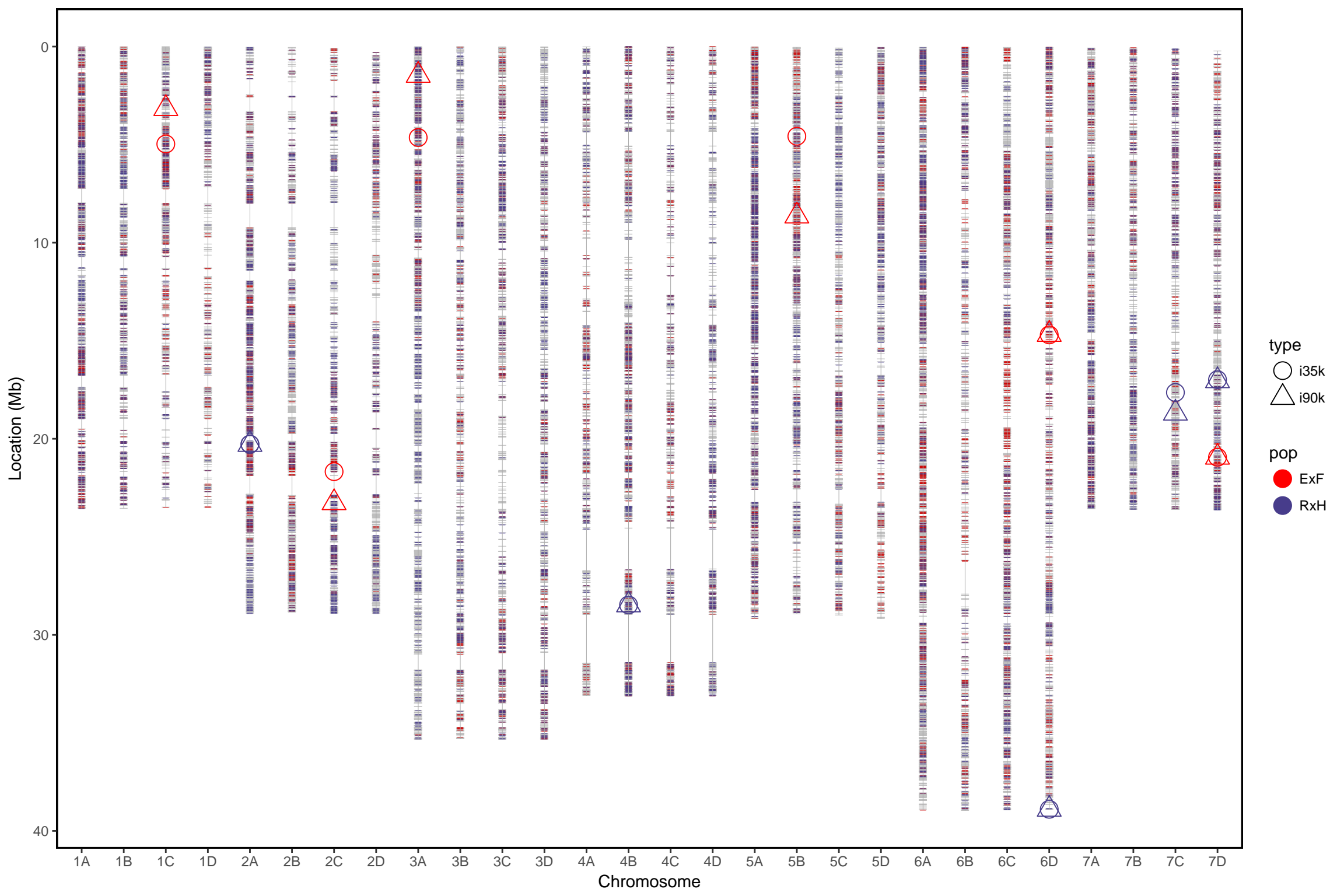

Supplement: Supplementary file 2 — Linkage map displaying marker positions (grey) in Mb for 28 linkage groups of octoploid strawberry (1A–7D) marker positions scaled to F. vesca genome. Markers overlapping between the validation set and ‘Emily’ x ‘Fenella’ and ‘Redgauntlet’ x ‘Hapil’ populations are red and blue ‘-’, respectively. QTL locations from combined analysis ‘Emily’ x ‘Fenella’ (red) and ‘Redgauntlet’ x ‘Hapil’ (purple) (PDF 263 kb) [file 122_2018_3128_MOESM2_ESM.pdf]
