# Supplementary material for: Identification of powdery mildew resistance QTL in strawberry (Fragaria × ananassa)
Source: Theor Appl Genet. 2018 Jul 3;131(9):1995–2007. doi: 10.1007/s00122-018-3128-0 (PMC6096635; doi:10.1007/s00122-018-3128-0)
Supplement: Supplementary file 3 — Supplementary material 3 (PDF 44 kb) [file 122_2018_3128_MOESM3_ESM.pdf]

Supplementary Table 1 Focal single nucleotide polymorphisms (SNP) linked with each quantitative trait loci associated with strawberry powdery mildew disease resistance identified through the Kruskal-Wallis analysis. Individual analysis is conducted for each phenotyping event. Dark grey shading highlights incidences where identical SNP have been identified. Light grey shading denotes SNP which represent the same QTL.

LG	Marker name	Position (Mb)	H	sig	Parent	Year
1C	Affx.88811774	3.6	21.1	*****	Emily	2012a
1C	Affx.88810959	0.8	24.3	******	Emily	2012b
1C	Affx.88809498	0.3	19.8	*****	Emily	2013a
1C	Affx.88813625	5.9	17.6	****	Emily	2013b
2A	Affx.88819993	10.6	6.8	**	Fenella	2012b
2A	Affx.88826282	20.3	10.8	***	Hapil	2012
2A	Affx.88826254	20.3	14.0	***	Hapil	2013
2A	Affx.88826254	20.3	20.6	*****	Hapil	2014
2B	Affx.88830297	28.4	9.4	**	Emily	2011
2C	Affx.88826973	21.5	15.6	****	Emily	2012b
2C	Affx.88879338	23.3	12.2	***	Emily	2013a
3A	Affx.88831545	1.5	5.4	*	Fenella	2012b
3A	Affx.88834579	4.6	7.1	**	Fenella	2013a
3C	Affx.88835425	8.4	15.3	****	Fenella	2013a
3C	Affx.88834576	4.6	7.7	**	Hapil	2012
3D	Affx.88846435	22.0	6.0	*	Fenella	2012b
3D	Affx.88835461	8.3	14.6	***	Emily	2013a
4A	Affx.88846914	0.1	10.3	**	Emily	2012b
4A	Affx.88846745	1.3	10.6	**	Emily	2013a
4B	Affx.88854428	24.2	10.4	**	Emily	2011
4B	Affx.88851752	32.5	10.2	*	Emily & Fenella	2014
4C	Affx.88848483	7.5	5.5	*	Hapil	2012
5A	Affx.88859525	1.7	11.2	***	Fenella	2013a
5B	Affx.88862333	8.6	17.8	****	Fenella	2012a
5B	Affx.88862333	8.6	15.1	***	Fenella	2012b
5B	Affx.88862333	8.6	8.9	**	Fenella	2013a
5B	Affx.88861172	4.5	20.5	*****	Fenella	2013b
5C	Affx.88864901	13.2	8.3	**	Fenella	2014
5D	Affx.88864140	9.9	11.3	***	Hapil	2012
6A	Affx.88882922	21.7	7.0	**	Emily	2013b
6B	Affx.88880140	31.2	9.1	**	Emily	2013b
6D	Affx.88880958	14.7	9.5	**	Fenella	2012a
6D	Affx.88880958	14.7	9.1	**	Fenella	2013a
6D	Affx.88883211	22.3	7.8	**	Fenella	2012b
6D	Affx.88904022	38.9	23.0	*****	Redgauntlet	2012
6D	Affx.88904022	38.9	12.8	***	Redgauntlet	2013
6D	Affx.88904022	38.9	15.9	****	Redgauntlet	2014
6D	Affx.88904022	38.9	13.7	***	Redgauntlet	2016
7D	Affx.88898584	17.0	12.5	***	Hapil	2013
7D	Affx.88902178	20.9	21.2	*****	Emily	2012b
7D	Affx.88902178	20.9	19.1	****	Emily	2013b
